# A Cross-Cultural Comparison of the Effects of Alcohol Mixed with Energy Drink (AMED) Consumption on Overall Alcohol Consumption and Related Consequences

**DOI:** 10.3390/ijerph18147579

**Published:** 2021-07-16

**Authors:** Sarah Benson, Sean J. Johnson, Chris Alford, Andrew Scholey, Joris C. Verster

**Affiliations:** 1 Centre for Human Psychopharmacology, Swinburne University, Melbourne, VIC 3122, Australia; sarahbenson@swin.edu.au (S.B.); andrew@scholeylab.com (A.S.); 2 Centre for Trials Research, Cardiff University, Cardiff CF14 4YS, UK; JohnsonS11@cardiff.ac.uk; 3 Department of Health and Social Sciences, University of the West of England, Bristol BS16 1QY, UK; chris.alford@uwe.ac.uk; 4 Nutrition Dietetics and Food, School of Clinical Sciences, Monash University, Melbourne, VIC 3168, Australia; 5 Division of Pharmacology, Utrecht Institute for Pharmaceutical Sciences (UIPS), Utrecht University, 3584CG Utrecht, The Netherlands

**Keywords:** alcohol, energy drink, alcohol consumption, consequences, risk taking

## Abstract

There is a growing body of scientific literature examining the effects of alcohol mixed with energy drink (AMED) on alcohol consumption and related negative consequences, such as risky behavior or negative health effects. It is unknown whether differences in cultural context may influence these AMED effects. The current cross-cultural study compared the data of N = 6881 students from The Netherlands (N = 4424), UK (N = 1594), and Australia (N = 863). Demographics, alcohol consumption, its consequences, and motives for AMED consumption were assessed. Analyses included (a) between-groups comparison of AMED and alcohol only (AO) consumers, (b) within-subjects comparison of AMED and AO occasions among AMED consumers only, and (c) comparisons between the three countries. The between-groups analysis revealed that AMED consumers drink more alcohol than AO consumers (*p* < 0.001). AMED consumers differed from AO consumers in many other aspects. For example, AMED consumers were significantly more often a smoker and had higher risk-taking scores. Within subject analysis among AMED consumers showed that significantly less alcohol was consumed on AMED, compared to AO occasions (*p* < 0.001). These findings were observed for both typical drinking occasions and the past month’s heaviest drinking occasion, and were consistent across the three countries. Comparisons between countries revealed that on both AMED and AO occasions, the UK sample consumed significantly more alcohol than the Australian and Dutch samples. Across countries, neutral motives such as ‘I like the taste’ and ‘I wanted to drink something else’ were the most frequently reported motives for consuming AMED. The most notable difference between the countries was the finding that consuming AMED ‘To get drunk’ was endorsed significantly more often among the UK sample (45.6%) than the Australian (31.2%) and Dutch (8.0%) samples. Negative alcohol-related consequences were significantly less frequently reported for AMED occasions compared to AO occasions. Some country-specific consequences of AMED consumption were observed, but these were more likely related to characteristics of the country and their drinking culture (e.g., the Australian sample reported more often driving a car after AMED consumption compared to the other countries, and this pattern of results was also found for AO occasions). In conclusion, there were limited differences between countries with regard to demographics of consumers and motives for AMED consumption, but the UK sample consumed significantly more alcohol and reported the highest frequency of negative alcohol related consequences. Consistent across countries was the observation that AMED consumers consume significantly less alcohol on their past month heaviest AMED drinking occasion, compared to their past month heaviest AO drinking occasion.

## 1. Introduction

Energy drinks are non-alcoholic beverages, typically containing 80 mg of caffeine per 250 mL serving size, and other functional ingredients such as B-vitamins, glucose and taurine. The consumption of energy drinks is popular [1] and may be related to the positive effects of energy drink on alertness and cognitive performance [2]. For example, it has been shown that the consumption of a 250 mL energy drink has the same positive effects on alertness and driving ability as a cup of coffee, both containing 80 mg of caffeine [3,4]. Across all age groups, the total caffeine amount ingested via energy drinks is a small fraction (<5%) of daily caffeine intake [5]. Instead, coffee, tea, and to a lesser extent, caffeinated soft drinks such as colas account for the majority of daily caffeine intake. In addition to the regular (i.e., unmixed) consumption of energy drinks, a minority of people sometimes mix energy drinks with alcohol [6].

Over the past decade, several studies have investigated the effects of consuming alcohol mixed with energy drink (AMED). Early research typically adopted a between-groups design, comparing AMED consumers with alcohol only (AO) consumers. These studies, usually conducted among university students, consistently reported that AMED consumers have higher levels of alcohol consumption compared to AO consumers. These findings resulted in concerns about mixing alcohol with energy drink, and the possible role of energy drinks in increasing alcohol consumption, reducing subjective intoxication, and increasing experiences of alcohol related negative consequences [6]. However, studies also found that AMED consumers differ in many aspects from people that do not consume AMED [6,7]. A recent review summarizing these differences found, for example, that AMED consumers were more frequently male, single, and a fraternity or a sorority member, and had increased levels of sensation seeking, smoking, drug use, and unsafe sex [6]. Given the overall observed differences in demographics and personality, Verster et al. [6] concluded that “The literature is overwhelmingly consistent with the notion that AMED consumption is just one manifestation of an underlying trait for greater alcohol consumption along with a cluster of other risky behaviors” (p. 15). In other words, it is likely that AMED consumers show the same high levels of alcohol consumption on AMED and AO occasions.

This was confirmed by subsequent research using within-subject designs which compared AMED and AO drinking occasions in AMED consumer cohorts. Meta-analyses combining the results of these studies showed that mixing alcohol with energy drink did not significantly increase overall alcohol consumption [6,8], did not significantly alter subjective intoxication [9], and did not increase the number of experienced negative alcohol-related consequences [6]. Thus, although AMED consumers usually drink more alcohol than AO consumers, this is observed irrespective of whether the drinking occasion concerns AMED or AO consumption.

These within-subject studies were conducted in various countries, including USA, Canada and Australia [10,11,12,13]. While the overall results showed no differences in alcohol consumption on AMED and AO occasions [6,8], there may be sociocultural differences between countries in the magnitude of the effects of AMED consumption on overall alcohol consumption. Therefore, it is important to investigate to what extent the overall results are consistent across countries and subpopulations. Drinking cultures vary considerably between countries, but also within countries (e.g., students versus the working population) [14]. Additionally, the popularity of alcohol (and mixing with energy drink) varies between countries and consumer groups [15]. Demographic, sociocultural, ethnic, and religious characteristics of societies have an impact on the frequency and quantity of alcohol consumption. In some countries, alcohol consumption is generally accepted as part of daily life, e.g., in combination with a meal (so-called ‘wet’ countries), whereas in other countries alcohol use is not common and is restricted (i.e., ‘dry’ countries). However, this broad distinction between drinking cultures oversimplifies cross-cultural differences [16]. For example, additional variation between countries may exist in beverage types, serving sizes, alcohol content, and timing of drinking. Additionally, the extent to which heavy alcohol consumption and drunkenness is socially accepted, the presence of peer pressure during drinking occasions, or the consumption of alcohol by women, varies between countries. In addition, differences in the accessibility of alcohol (e.g., selling points, drinking venues, and their opening and closure times), and pricing may affect the frequency and quantity of alcohol consumption. Further, differences between countries in the advertisements for alcohol or energy drink consumption versus media or governmental attention for potential harm of their consumption may affect quantity, frequency, and popularity of these beverages. It is understandable that such sociocultural differences may have an impact on the amount of alcohol and AMED consumed in different countries.

Previous research suggests that cross-cultural differences may exist. For example, in the USA, Woolsey et al. [10] reported a reduction of 2.3 alcoholic drinks on regular AMED versus AO occasions, and a reduction of 7.4 alcoholic drinks on the past year’s heaviest AMED occasion (10.6 alcoholic drinks) compared to the past year’s heaviest AO occasion (18.2 alcoholic drinks). In contrast, in Canada, Brache and Stockwell [11] reported an increase of 0.8 alcoholic drinks on AMED occasions. It should be taken into account, however, that a direct comparison between countries is needed to be able to ascribe the observed difference in drinking behavior to cross-cultural differences. In fact, it is likely that discrepancies and limitations of the different study designs may be responsible for the inconsistent findings between the two countries. The study designs (e.g., survey versus on premise) and included convenience samples (e.g., athletes vs. students) and sample size are different between the studies. Time periods of assessment also differ (e.g., past year versus past 30 days), as do the (absence of) descriptions of AMED, and the wording of questions asked to participants. The discrepancy in study outcomes illustrates the importance of employing identical study designs if one aims to conduct a comparison between countries. However, when studies are conducted in the same country cross cultural differences may also be present. For example, in Australia, survey research by Peackock et al. [12] found an increase of 0.6 alcoholic drinks on AMED occasions in Tasmania, whereas on-premise interviews by Lubman et al. [13] reported a reduction of 0.7 alcoholic drinks on AMED occasions in New South Wales. Taken together, a direct comparison of AMED consumption between or within countries, using the same methodology, is needed for a valid and reliable interpretation of the data on possible cross-cultural differences.

To date, there has been no direct comparison of cross-cultural differences in drinking behavior that may have an impact on AMED consumption, using the same methodology and design. Therefore, the purpose of the current study was to compare data from three identical survey studies using a within subjects-design, conducted in three different counties (the Netherlands, UK, and Australia) [17,18,19]. It was hypothesized that, due to variability in drinking cultures, motives, frequency and quantity of alcohol consumption, and consequences of drinking may differ between the three countries.

## 2. Materials and Methods

### 2.1. Design

An online survey was designed to investigate the impact of mixing alcoholic beverages with energy drinks on overall alcohol consumption and alcohol-related consequences among Dutch, UK and Australian students [17,18,19]. In all three countries the same survey was conducted to enable a cross-cultural comparison of the study outcomes [20].

The surveys were completed online via SurveyMonkey^®^ (Palo Alto, CA, USA). Informed consent was obtained electronically at the introductory page of the survey, which also provided a short description of the survey’s purpose and content, and information concerning anonymity and data handling.

### 2.2. Recruitment

In the Netherlands, all 70,000 students of Utrecht University and Utrecht University of Applied Sciences were contacted via e-mail to complete the survey. UK students were approached via university student unions (England, Wales, Scotland, Northern Ireland) (N = 139) who advertised the survey via their social media platforms. The Australian students were recruited via word of mouth, advertisements on social media (i.e., Facebook) and flyers.

### 2.3. Demographics

Participants were asked to indicate their sex, age, weight, and height. Furthermore, smoking status, illicit drug use, and the use of medicinal drugs were assessed.

### 2.4. Risk Taking

The RT-18 questionnaire [21] was completed to assess the subjects’ level of risk-taking behavior. The 18 items can be answered with ‘yes’ or ‘no’ (0 or 1 point, depending on the item), and the sum score of the RT-18 ranges from 0 (no risk-taking) to 18 (extreme risk-taking). In addition, two subscales can be derived: (1) risk-taking (engagement in risk-taking behavior), and (2) risk assessment (the level of awareness of the consequences of one’s risk-taking behavior).

### 2.5. Alcohol Consumption

Alcohol consumption questions focused on beverage consumption in the past 30 days. These questions were adapted from the Quick Drinking Screen [22,23]. Questions comprised past month’s quantity of alcohol consumption on a typical drinking occasion, the number of alcohol consumption days, number of days drunk, and the number of binge drinking days (i.e., more than 4 (women) or 5 (men) alcoholic drinks consumed). Guidance was provided regarding the standardized size of alcoholic drinks using pictures of different serving sizes (e.g., glass, shot, bottle) along with the content in ml, and how to transfer common amounts (e.g., a bottle of wine) into standard units (of 10 g pure alcohol in each country). The term ‘unit’ and ‘drink’ are used interchangeably in the remainder of this article. AMED consumers answered these questions for both AMED and AO drinking occasions. For AMED occasions, the number of energy drinks (standard serving size of 250 mL) mixed with alcohol was also assessed. Mixing was defined as consuming an energy drink within a time period of +/− 2 h of alcohol consumption, which represents a conservative definition of ‘mixing’ [20]. Finally, for the past month’s heaviest AMED and AO occasion, the number of energy drinks and alcoholic drinks were recorded, and the duration of the drinking session.

### 2.6. Motives for AMED Consumption

AMED consumers were asked to indicate their motives for consuming AMED. The motives included ‘I like the taste’, ‘I wanted to drink something else’, ‘I felt sad’, ‘To get drunk’, ‘To prevent getting drunk’, ‘It feels like I can drink more alcohol’, ‘It feels like energy drinks reduce the negative effects of alcohol’, ‘To sober up’, ‘To celebrate a special occasion, party’, ‘Because others drink it as well’, ‘I received the drink from someone else (and did not want to refuse it)’, ‘To make me happy’, and ‘To prevent next day hangover’. Participants indicated whether or not each motive applied to them.

### 2.7. Alcohol-Related Consequences

To evaluate the alcohol-related negative consequences associated with AMED and AO drinking occasions, the Brief Young Adult Alcohol Consequences Questionnaire (BYAACQ) was completed [24,25]. The BYAACQ consists of a listing of 24 possible consequences of alcohol consumption. The items can be answered with ‘yes’ or ‘no’, depending on whether or not the statement was applicable to the participant within the past year. The BYAACQ was completed by AMED consumers for both AO and AMED occasions.

### 2.8. Statistical Analyses

Data were analyzed using IBM SPSS Version 27 (IBM SPSS Statistics 2019 Armonk, NY, USA: IBM Corp.). For the current analyses, participants were included if they consumed alcohol during the past month. The data of two groups were evaluated for each country: a sample of AMED consumers (consuming both AMED and AO), and a sample of participants who consumed alcohol only (consuming AO). Mean and standard deviation (SD) or the percentage of endorsement was computed for all variables, and the distribution of the data was checked for normality. First, the demographic data of AMED and AO consumers were compared using independent t-tests (for normally distributed and continuous data) or the Independent Samples Mann–Whitney U Test for data that was not normally distributed. Percentages between the groups were compared using Chi-squared tests. Second, cross-cultural comparisons of motives for consuming AMED were made. Paired comparisons of the reported percentages for different motives for AMED consumption between the three countries were conducted with the Chi-squared test. Third, alcohol consumption variables between the countries, or between AMED and AO consumers were compared. The alcohol consumption data were not normally distributed; therefore, these between-group comparisons were made using the Independent Samples Kruskal–Wallis test. If the main effect was statistically significant, post hoc pairwise comparisons were conducted, applying appropriate Bonferroni correction to account for multiple comparisons. AMED consumers who reported that they consumed AMED on their past months heaviest drinking occasion but did not report consuming any energy drinks on this occasion were excluded. Additionally, data on energy drink consumption from three UK participants that reported an unreliably high number of energy drinks on their heaviest drinking occasion (≥30 cans of 250 mL energy drink) were omitted from the analysis. Fourth, alcohol consumption of AMED consumers on AMED and AO occasions was evaluated. These within-subject comparisons were conducted with the Related Samples Wilcoxon Signed Rank Test. Comparisons between the countries were made using the Independent Samples Kruskal–Wallis test. If the main effect was statistically significant, post hoc pairwise comparisons were conducted, applying appropriate familywise Bonferroni correction to account for multiple post hoc comparisons between the countries, by multiplying the original *p*-value by the number of paired comparisons and comparing the adjusted *p*-value with the original cut-off value for significance (*p* < 0.05). Finally, the percentages of endorsed alcohol-related consequences associated with AMED or AO occasions (within-subjects) were evaluated. Overall BYAACQ scores for AMED and AO occasions were compared using the Related Samples Wilcoxon Signed Rank Test, and comparisons of these scores between countries were conducted using the Independent Samples Kruskal–Wallis Test (two-sided, with Bonferroni correction). Paired comparisons of individual (binary) BYAACQ item scores of AMED and AO occasions were conducted using the McNemar test (two-sided). Pairwise comparisons between the three countries were conducted with the Chi-squared test. Differences were considered statistically significant if *p* < 0.05.

## 3. Results

### 3.1. Demographics

In the Netherlands N = 3185 AO consumers (i.e., they consumed alcohol but did not mix alcohol with energy drinks) and N = 1239 AMED consumers (i.e., they consumed both AO and alcohol with energy drinks) completed the survey. In the UK, N = 865 AO consumers and N = 729 AMED consumers completed the survey. In Australia, N = 629 AO consumers and N = 234 AMED consumers completed the survey. Sample demographics are summarized in Table 1.

The data show that, across countries, AMED consumers were younger than AO consumers (*p* < 0.001), more often a smoker (*p* < 0.001) and reported using illicit drugs more frequently (*p* < 0.001). Additionally, the onset age of first time and regular alcohol consumption was younger than that of AO consumers (*p* < 0.001). Most notably, significantly greater expressions of these types of risk-taking behaviors was reflected in significantly higher RT-18 risk-taking scores among AMED consumers (*p* < 0.001 for the overall score, and factors 1 [risk-taking)] and 2 [risk-assessment]).

With regard to differences between the countries, reporting smoking tobacco was significantly more common in the Dutch sample, both among AMED and AO consumers (all comparisons *p* < 0.0017), and lowest in the Australian sample (*p* < 0.001). Past year illicit drug use did not differ between The Netherlands and Australia, and the percentages of past year illicit drug users among AMED consumers in these countries were significantly higher than in the UK. The percentage of medication users did not differ significantly between AMED consumers of the three countries. However, compared to the Netherlands, the percentage of medication users among AO consumers was significantly lower in the UK (*p* = 0.0017). The reported age of first alcohol consumption of both AMED and AO consumers was younger in UK, and older in Australia. For the reported age of regular alcohol consumption, Australian participants were significantly older than their Dutch and UK peers (*p* < 0.001). No significant difference in reported age of regular alcohol consumption was found between Dutch and UK students. Finally, among both AMED and AO consumers, Australian participants scored significantly higher on risk-taking than students from the UK and the Netherlands (*p* < 0.001).

### 3.2. Motives for AMED Consumption

Table 2 summarized the motives for consuming AMED.

Across countries, neutral motives such as ‘I like the taste’ and ‘I wanted to drink something else’ were the most frequently reported reasons for AMED consumption. Overall, other motives were endorsed by less than a quarter of participants. With regard to cross-cultural differences, Dutch students reported significantly more often that they liked the taste of AMED, but significantly less often that they consumed AMED to celebrate a special occasion or to party compared to the UK and Australian samples. In the Netherlands, peer pressure seems less dominant compared to the UK and Australia, as Dutch students significantly less often ‘received the drink from someone else and did not want to refuse it’ or consumed AMED ‘because others drink it as well’.

Drinking AMED ‘To get drunk’ was endorsed significantly more often among UK and Australian participants compared to the Dutch sample (*p* < 0.001). Finally, although the overall percentages are relatively low (<10%), Australian students were more likely to consume AMED because ‘it feels like it reduces the negative effects of alcohol’, or because it feels like they can drink more alcohol.

### 3.3. Usual Alcohol Consumption: Between-Group Comparisons of AMED and AO Consumers

Table 3 summarizes the between-group comparison of alcohol consumption of AMED and AO consumers on AO occasions. It is evident from Table 3 that, on both the usual and heaviest AO drinking occasions, AMED consumers drink significantly more alcohol than AO consumers (*p* < 0.001). Additionally, on the heaviest drinking occasion, the duration of alcohol consumption is significantly longer in the AMED group compared to the AO group (*p* < 0.001). This observation is consistent across the three countries.

### 3.4. Usual Alcohol Consumption: Within-Subject Comparisons among AMED Consumers

Table 4 summarizes the within-subject comparisons among AMED consumers comparing reported alcohol consumption on AMED and AO occasions. Overall, AMED consumers reported drink significantly more alcohol (*p* < 0.001), and more frequently on AO occasions compared to AMED occasions (*p* < 0.001).

When comparing the countries, UK AMED consumers appeared to be the heaviest drinkers, both on AMED and AO occasions. Compared to the Dutch sample, on both AMED and AO occasions UK AMED consumers reported significantly more drinking days (*p* < 0.001), more days drunk (*p* < 0.001) and more binge drinking days (*p* < 0.001). On usual AO occasions, the number of alcoholic drinks consumed was significantly higher among the UK sample. No significant difference in alcohol intake on usual AMED drinking occasions was observed between the Dutch and UK sample (*p* = 0.354). Compared to Australian drinkers, on AO occasions the UK drinkers reported consuming significantly more alcohol (*p* < 0.001), reported more days drunk (*p* = 0.001) and more binge drinking days (*p* = 0.025). Additionally, on AMED occasions, compared to Australian drinkers, the UK drinkers reported consuming significantly more alcohol (*p* < 0.001), reported more days drunk (*p* < 0.001), and more binge drinking days (*p* < 0.001).

Compared to the Dutch sample, the Australian sample reported significantly more days drunk (*p* < 0.001) and more binge drinking days (*p* < 0.001). No significant differences between the two countries were found for the number of alcoholic drinks consumed on usual AMED occasions (*p* = 0.305) and the number of AMED drinking days (*p* = 0.679). For AO occasions, no significant differences between the two countries were found for number of alcoholic drinks consumed (*p* = 0.963) or the number of binge drinking days (*p* = 0.132). The Dutch sample reported significantly more alcohol consumption days than the Australian sample (*p* < 0.001), whereas the Australian sample reported significantly more days being drunk (*p* < 0.001).

### 3.5. Alcohol Consumption of AMED Consumers on Their Heaviest AMED and AO Drinking Occasions

The data of the within-subjects analysis of the past months heaviest AMED and AO drinking occasions are presented in Table 5. Overall, significantly more alcohol was consumed on the past month’s heaviest AO drinking occasion compared to the past month’s heaviest AMED drinking occasion (*p* < 0.001), and the drinking duration was significantly longer for AO (*p* < 0.001). The UK sample reported consuming significantly more alcohol (*p* < 0.001) on their heaviest AO drinking occasion compared to the Australian sample, but no significant differences in drinking duration were found. On their heaviest AMED drinking occasion, the UK and Australian samples did not significantly differ in the number of reported alcoholic drinks consumed (*p* = 0.113) or the duration of the drinking occasion (*p* = 1.000).

Compared with the Dutch sample, the UK sample reported consuming more alcohol on both the AMED and AO heaviest drinking occasion (*p* < 0.001 and *p* < 0.001, respectively). No significant differences in drinking duration were found.

Compared to the Dutch sample, the Australian sample did not report consuming more alcohol on the AO occasion (*p* = 0.068); however, the Dutch sample reported a significantly longer duration of drinking (*p* = 0.033). No significant differences in reported alcohol intake and drinking duration were found for the AMED occasion.

### 3.6. Number of Energy Drinks Mixed with Alcohol

**Figure 1:**
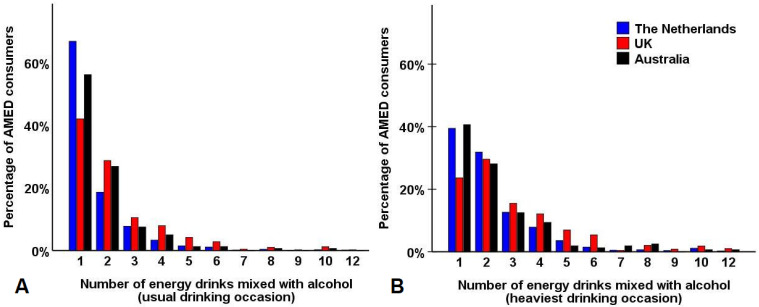
Number of energy drinks mixed with alcohol. Shown are the number of energy drinks mixed with alcohol on (**A**) a usual drinking occasion; (**B**) past month’s heaviest drinking occasion.

On average, participants reported a mean (SD) of 1.7 (2.2) AMED consumption days per month, and on these days, they mixed 1.8 (1.8) energy drinks with alcohol. The distribution of the number of energy drinks mixed with alcohol on usual and heaviest AMED consumption occasions is depicted in Figure 1. For the majority of AMED consumers, the number of energy drinks mixed with alcohol on a usual AMED occasion was one 250 mL can (57.8%) or two 250 mL cans (22.7%). Consuming three cans was reported by 9.2% of respondents, whereas 10.3% reported consuming more than three energy drinks on usual AMED occasions (see Figure 1A). On their heaviest AMED drinking occasion, the most frequently reported number of energy drinks mixed with alcohol was one 250 mL can (34.2%) or two 250 mL cans (30.7%). Consuming three cans was reported by 13.6% of respondents, whereas 21.6% reported consuming more than three energy drinks on their heaviest AMED drinking occasion (see Figure 1B). On average, across all countries participants consumed 2.6 (2.2) 250 mL cans of energy drink on their heaviest drinking occasion.

On usual AMED drinking occasions (see Table 4), the UK sample reported consuming significantly more energy drinks than the Australian sample (*p* < 0.001) and the Dutch sample (*p* < 0.001). The Dutch sample reported consuming significantly more energy drinks mixed with alcohol on usual AMED drinking occasions compared to the Australian sample (*p* = 0.006). On the heaviest AMED drinking occasion (see Table 5), the UK sample reported consuming significantly more energy drinks than the Australian sample (*p* = 0.004) and the Dutch sample (*p* < 0.001). No significant difference in the reported number of energy drinks mixed with alcohol on their heaviest AMED drinking occasion was found between the Dutch and Australian samples.

### 3.7. Alcohol-Related Consequences

The alcohol-related consequences of consuming AMED or AO, reported by AMED consumers from the three countries, are summarized in Table 6.

Overall, all alcohol related consequences were significantly more frequently reported for AO occasions compared to AMED occasions. For the Netherlands and the UK, all consequences were reported significantly more frequently on AO occasions. For the Australian sample, all consequences were experienced significantly more frequently on AO occasions, excluding the items “I have felt like I needed a drink after I’d gotten up (that is, before breakfast)” (*p* = 1.000), “I have been overweight because of drinking” (*p* = 1.000), “I have become very rude, obnoxious, or insulting after drinking” (*p* = 0.151).

In line with the significantly higher alcohol consumption levels of UK drinkers, compared to the Dutch sample, their overall BYAACQ scores for AO and AMED occasions were also significantly higher (*p* < 0.001). Additionally, for both AO and AMED occasions all individual alcohol-related consequences were significantly more frequently reported by the UK sample.

In contrast, no significant differences in overall BYAACQ scores were found between the UK and Australian sample for either AO occasions (*p* = 0.984) or AMED occasions (*p* = 0.617). Compared to the Australian sample, all individual BYAACQ items were significantly more frequently reported by UK drinkers, excluding the item “I have driven a car when I knew I had too much to drink to drive safely” which was, for both AO and AMED occasions, more frequently endorsed by the Australian sample. For AMED occasions, no significant difference between the UK and Australian sample was found for the item ‘My physical appearance has been harmed by my drinking’ (*p* = 0.063).

Compared to the Dutch sample, the Australian sample had a significantly higher overall BYAACQ score on both AO and AMED occasions (*p* < 0.001). For AO occasions, the Australian sample reported significantly higher rates of all individual alcohol-related consequences, except the items ‘I have had a hangover (headache, sick stomach) the morning after I had been drinking’ (*p* = 0.099), ‘I have had less energy or felt tired because of my drinking’ (*p* = 0.144), ‘I have not gone to work or missed classes at school because of drinking, a hangover or illness caused by drinking’ (*p* = 0.062), and ‘I have been overweight because of drinking’(*p* = 0.181). For AMED occasions, the Australian sample reported significantly higher rates for all individuals item of the BYAACQ compared to AO.

### 3.8. Sex Differences

For the whole sample, analyses were conducted to evaluate possible differences between men and women. Demographic data of both sexes are summarized in Table 7.

Among AMED consumers, men significantly more often reported using illicit drugs (*p* < 0.001), and significantly less often reported using medicinal drugs than women (*p* < 0.001). Men were taller (*p* < 0.001) and heavier (*p* < 0.001) than women, and had significantly higher risk-taking scores than women (*p* < 0.001). No sex differences were found regarding smoking status or age of onset of alcohol consumption. Among AO consumers, men were older (*p* < 0.001), taller (*p* < 0.001) and heavier (*p* < 0.001) than women. Past year illicit drug use was more frequently reported by AO men, whereas medicinal drug use was more frequently reported by AO women. AO men had significantly higher (*p* < 0.001) risk-taking scores than AO women, and started consuming alcohol regularly at a younger age (*p* < 0.001). In both men and women, differences between the AO group and AMED group were significant (*p* < 0.001 for all comparisons, except *p* = 0.027 for weight among women).

Table 8 summarized the motives for consuming AMED among men and women. With the exception of ‘I like the taste’, ‘To celebrate a special occasion’, ‘To prevent getting drunk’ and ‘I felt sad’, men endorsed all items significantly more frequently than women (Table 8) (*p* < 0.05). In particular, negative motives for consuming AMED such as ‘To get drunk’ and ‘It feels like I can drink more alcohol’ were significantly more often reported by men than women (*p* < 0.05).

Table 9 summarizes alcohol consumption of AMED and AO consumers. The between-group comparisons reveal that in both men and women AMED consumers reported greater quantity and frequency of alcohol consumption compared to the AO group (*p* < 0.001). In both men and women, all differences between the AMED and AO group were significant, (*p* = 0.029 for drinking days per week among men; *p* < 0.001 for all other comparisons).

Table 10 summarizes the within-subject comparisons among AMED consumers comparing reported alcohol consumption on AMED and AO occasions. For both men and women, AMED consumers reported drinking significantly more alcohol (*p* < 0.001), and more frequently on AO occasions compared to AMED occasions (*p* < 0.001). For all assessments, the quantity and frequency of alcohol consumption was significantly higher in men than women (*p* < 0.001).

Finally, Table 11 summarizes the percentage of negative alcohol-related consequences reported for AMED and AO occasions. For both men and women, negative consequences were significantly more frequently reported for AO occasions compared to AMED occasions. This observation was expected as it is in line with the fact that AMED occasions are significantly less frequent than AO occasions, and that significantly less alcohol is consumed on AMED occasions compared to AO occasions (See Table 10). In line with the observation that women consume less alcohol (both in terms of frequency and quantity) than men (see Table 10), women reported significantly fewer negative alcohol-related consequences than men. The latter was evident on both AMED and AO occasions.

## 4. Discussion

The aim of this study was to conduct a cross-cultural comparison of the motives, consumption levels, and consequences of consuming AMED, applying both between-group comparisons with AO drinkers and within-subject comparisons between AMED and AO occasions.

The between-group analysis revealed that AMED consumers reported drinking more alcohol than AO consumers on both AMED and AO drinking occasions. Comparing the characteristics of the two groups reveals that AMED consumers score significantly higher on risk taking, which may be an explanation for this observation. These higher levels of risk-taking behavior among AMED consumers likely contribute to the higher alcohol intake levels on any drinking occasion, irrespective of whether alcohol was consumed alone or mixed with energy drink. In line with this, AMED consumers also reported higher levels of other health-related risk behaviors, such as smoking and illicit drug use. These differences between AMED and AO consumers are consistently observed, independent of country or sex.

Within-subject comparisons of AMED and AO occasions among AMED consumers, however, show that AMED users reported consuming significantly more alcohol on AO than AMED occasions. Additionally, alcohol-related negative consequences were significantly less often reported for AMED drinking occasions. This observation is not unique to the datasets analyzed in this paper. For example, similar results were found in samples from USA [6] and Australia [13]. Together, these findings support the hypothesis that the co-consumption of energy drinks is one of the many expressions of a high risk-taking type of lifestyle and personality [6,7].

It is not clear why significantly less alcohol is consumed on AMED occasions compared to AO occasions. One could speculate that this may be related to general drinking behaviors (e.g., one is used to drinking only one type of drink, which is only infrequently changed for an alternative beverage). This idea is supported by our data, showing that, although participants consume considerable amounts of alcohol on usual and past month’s heaviest drinking AMED occasions, only about one third of these drinks are mixed with energy drink (32.1% on usual drinking occasions, and 35.1% on their past month heaviest drinking occasion). In fact, 80.5% of respondents report energy drink consumption of one or two 250 mL cans on a typical AMED drinking occasion, which corresponds to caffeine intake levels of 80 mg to 160 mg, which are considered safe by legislative bodies, such as the European Food Safety Authority (EFSA), who suggest an upper limit of 200 mg per consumption occasion [26]. An alternative possibility is that AMED is more likely to be consumed when individuals are fatigued based on the false premise that Energy Drinks may “mask” the depressant effects of alcohol. Despite overwhelming evidence to the contrary [9], this belief is still widely held and included in some scientific literature, largely as the result of one widely mis-reported study [27,28]. Finally, the fact that often energy drinks are often more expensive than other mixers may also play a role in selecting drink types.

Unique beverage characteristics may be responsible for the prevention of excessive consumption of energy drinks (e.g., its caffeine content or taste), but more research is needed to elucidate this possibility. An alternative explanation may be that, when there are significantly more AO drinking occasions compared to AMED drinking occasions, this increases the likelihood of a high drinking level for AO occasions. On the other hand, the observation is consistently found across studies and countries, including different study designs, and for assessment periods for up to one year. The consistent findings of the three studies, supported by meta-analyses [6,8], do give us some confidence that alcohol intake is relatively lower on AMED occasions compared to AO occasions. This finding needs to be confirmed, however in appropriately designed prospective studies where participants are matched for dispositional factors, such as risk-taking.

The most frequently endorsed motives for consuming AMED were of ‘neutral’ nature and comprised ‘I like the taste’ (the Netherlands), ‘I wanted to drink something else’ (UK), and ‘To celebrate a special occasion’ (Australia). Negative motives were less frequently endorsed (usually < 10%). An exception was consuming AMED ‘To get drunk’, which was endorsed by 45.64% of UK students and 31.2% of Australian students. These high endorsements were not seen in the Dutch sample (8.0%).

Regarding the negative consequences of alcohol consumption, across countries these were more frequently reported for AO occasions than for AMED occasions. One could argue that this difference may have been caused by the fact that participants have significantly more AO occasions than AMED occasions. However, the assessment period was rather long (i.e., past year) which makes it reasonable to assume that there were a sufficient number of both heavy drinking AMED and AO occasions to allow for a fair comparison of whether negative alcohol-related consequences were experienced at least once (or not). With regard to differences between countries, the data on negative consequences of alcohol consumption are consistent with those on the relative amounts of alcohol consumed across countries. For both AMED and AO occasions, negative consequences were most frequently reported by UK students, followed by Australian students and finally the Dutch sample. Two exceptions were observed concerning negative consequences that were most frequently reported by the Australian sample. The first item was ‘I have driven a car when I knew I had too much to drink to drive safely’. The reason for this is unknown but it may be a factor of typical transport use. Previous research shows that a significant percentage of Australian students use cars as their selected travel mode [29,30]. In contrast, in The Netherlands most students ride a bicycle [31]. A second item that was more frequently reported by the Australian sample was ‘I have felt like I needed a drink after I’d gotten up (that is, before breakfast)’. For this item, we have no specific reason why differences between the countries were observed. However, it should be taken into account that this item was the least frequently endorsed of all 24 negative consequences that were assessed (2.3–8.6%).

Scientific literature reveals that men consume more alcohol than women, on both AMED and AO occasions. In addition, in both men and women, alcohol consumption on AMED occasions was significantly lower compared to the amount of alcohol consumed on AO occasions [6,7,8]. These consistent findings were also found in the current study. Men also reported significantly higher risk-taking scores than women. As a possible consequence, compared to women, men reported experiencing more negative alcohol-related consequences, on both AMED and AO occasions. However, consistent among men and women, negative alcohol-related consequences were significantly less frequently reported for AMED occasions compared to AO occasions. With regard to motives for AMED consumption, some sex differences were observed. Most notably, compared to women, men significantly more often reported consuming AMED because they ‘wanted to drink something else’, and ‘to get drunk’. Taken together, most of the observed sex differences are related to the quantity and frequency of consuming alcohol per se, irrespective of whether alcohol is mixed with energy drink or not.

There are several limitations that need to be addressed. First, the data were collected retrospectively. Hence, recall bias may have played a role and influenced the accuracy of the collected data. To overcome this, prospective studies should be conducted with real-time assessments of alcohol and energy drink consumption and reporting of negative consequences. It is unclear to what extent recall bias has had an impact in the current studies. Primarily, we compared AMED and AO occasions, and there is no reason to assume that there is a difference in recall for these occasions, other than AO occasions being more frequent. As ‘usual’ drinking occasions may be an estimated average, the past month heaviest drinking occasion is likely an event that is well remembered. It is also likely that possible negative alcohol-related consequences were similarly remembered. There is no reason to assume that the level of recall bias would be different across the three counties where the studies were performed.

A second limitation is the fact that this paper is restricted to student samples, and that the UK the sample was national, compared to the Dutch sample, which was regional. A comparison of AMED and AO drinking behaviors according to student status has revealed that, at least in Australia, non-students consistently consume more alcohol and are involved in a greater number of negative alcohol-related consequences than their student counterparts [19]. Additionally, although the age range of 18 to 30 years old comprises the majority of AMED consumers, it remains to be determined if the current outcomes can be generalized to older or younger participants. Therefore, the current study should be replicated in other age groups and non-student samples. Third, in the surveys, we assessed motives for consuming AMED. Unfortunately, motives for alcohol consumption per se (AO, unmixed) were not assessed in the current study. Therefore, we could only compare the frequency of endorsement between the countries, but not establish whether motives for AMED and AO consumption are different from each other. However, motives for mixing alcohol with other beverages (AMOB), such as cola or tonic, have been published for the Dutch and UK samples [32,33]. The absence of large differences between motives for consuming AMED and AMOB suggest that energy drinks are not unique in comparison to other more common mixers. Fourth, we assessed whether alcohol-related negative consequences were experienced during the past year for AMED and AO comparisons. As items of the BYAACQ are answered with ‘yes’ or ‘no’, we were unable to assess the frequency of occurrence of these consequences. The latter is, however, of importance. Over the year there are significantly more AO occasions than AMED occasions. It is, therefore, possible that this may have biased the outcome. On the other hand, participants consumed significantly more alcohol on AO heaviest drinking occasions, making it more likely that they experienced one or more negative consequences compared to their AMED heaviest drinking occasions, as increased levels of consumption and intoxication are associated with more negative consequences. Finally, it must be noted that, similar to previous AMED research, the current research was conducted in convenience samples. Such cohorts are necessarily self-selecting and may not be as representative of the general population as truly random samples. It is recommended that future studies are conducted in nationally representative samples.

## 5. Conclusions

Overall, the data suggest that AMED consumers drink less alcohol and experience fewer negative alcohol-related consequences on AMED occasions than on AO occasions. This observation was consistent across the three countries, and in line with previous research. With regard to cross cultural differences, it was found that UK students consume more alcohol and AMED than their Dutch and Australian peers. Limited differences in motives for AMED consumption were found between the countries. Some country-specific consequences of AMED consumption were observed, but these were more likely related to characteristics of the country than to their drinking culture. Finally, for both AMED and AO consumers, the overall reported amount of alcohol consumed on heaviest drinking occasions is high and should be regarded as a public health concern.

## Figures and Tables

**Table 1:** Demographics.

Demographics	Overall (N = 6881)		The Netherlands (N = 4424)		UK (N = 1594)		Australia (N = 863)	
	AMED	AO	AMED	AO	AMED	AO	AMED	AO
N	2202	4679	1239	3185	729	865	234	629
Male/female ratio (%)	41.0/59.0 (±9.6)	32.1/67.9 (±9.2) *	39.1/60.9 (±9.6)	32.5/67.5 (±9.2) *	45.9/54.1 (±9.8) ^γ^	33.1/66.9 (±9.2) *	36.3/63.7 (±9.4) ^‡^	28.6/71.4 (±8.9) *
Age (years)	21.3 (2.6)	22.1 (3.3) *	21.5 (2.3)	22.1 (2.6) *	20.6 (2.0)	21.0 (2.3) *^γ^	22.0 (4.2) ^†‡^	23.5 (5.9) *^†^
Height (m)	1.74 (0.1)	1.73 (0.1) *	1.76 (0.1)	1.75 (0.1) *	1.73 (0.2)	1.69 (0.2) ^γ^	1.70 (0.1) ^†‡^	1.68 (0.2) ^†^
Weight (kg)	71.0 (15.3)	68.1 (12.7) *	70.4 (12.3)	68.9 (11.8) *	72.6 (19.4) ^γ^	66.2 (14.7) *^γ^	69.4 (15.8) ^‡^	66.9 (13.9) *^†^
Current smoker (%)	21.7 (±8.1)	10.7 (±6.1) *	41.7 (±9.7)	23.2 (±8.3) *	25.8 (±8.6) ^γ^	18.2 (±7.6) *^γ^	15.8 (±7.1) ^†‡^	10.8 (±6.1) *^†‡^
Past year illicit drug use (%)	33.4 (±9.2)	20.9 (±8.0) *	38.3 (±9.5)	22.0 (±8.1) *	24.9 (±8.5) ^γ^	17.8 (±7.5) *^γ^	34.2 (±9.3) ^‡^	19.2 (±7.7) *
Medication use (%)	21.6 (±8.1)	22.5 (±8.2)	23.0 (±8.2)	23.2 (±8.3)	19.5 (±7.8)	18.2 (±7.6) ^γ^	20.9 (±8.0)	25.1 (±8.5) ^‡^
Age first consumed alcohol	14.0 (2.4)	14.5 (2.3) *	14.0 (1.9)	14.5 (2.0) *	13.9 (3.0)	14.1 (3.0) ^γ^	14.6 (2.7) ^†‡^	15.1 (2.9) *^†‡^
Age regular alcohol use	16.8 (1.7)	17.3 (2.0) *	16.5 (1.7)	17.2 (1.9) *	17.0 (1.6) ^γ^	17.2 (1.9) *	17.4 (2.0) ^†‡^	18.1 (2.5) *^†‡^
RT18 total risk-taking score	7.9 (4.1)	6.1 (3.8) *	7.2 (4.0)	5.4 (3.6) *	8.4 (4.1) ^γ^	7.1 (3.9) *^γ^	9.9 (3.9) ^†‡^	8.5 (3.1) *^†‡^
RT18 Factor 1 (risk-taking)	4.9 (2.7)	3.8 (2.6) *	4.7 (2.6)	3.6 (2.5) *	5.3 (2.8) ^γ^	4.5 (2.8) *^γ^	5.4 (2.7) ^†^	4.2 (2.7) *^†^
RT18 Factor 2 (risk assessment)	2.9 (2.3)	2.3 (2.0) *	2.6 (2.2)	1.8 (1.9) *	3.1 (2.2) ^γ^	2.6 (1.9) *^γ^	4.5 (1.5) ^†‡^	4.3 (1.3) *^†‡^

Percentages and 95% confidence interval (between brackets) or mean and standard deviation (SD, between brackets) are shown. Significant differences between AMED and AO (adjusted *p* value < 0.05, after Bonferroni’s correction for multiple comparisons) are indicated by *, between the Netherlands and the UK by ^γ^, the Netherlands and Australia by ^†^, and between the UK and Australia by ^‡^. Abbreviations: AMED = alcohol mixed with energy drink; AO = alcohol only.

**Table 2:** Motives for alcohol mixed with energy drink (AMED) consumption.

Motives	Overall	The Netherlands	UK	Australia
I like the taste	74.4 (±8.6)	81.1 (±7.7)	66.5 (±9.3) ^γ^	64.2 (±9.4) ^†^
I wanted to drink something else	31.9 (±9.1)	35.3 (±9.4)	25.1 (±8.5) ^γ^	35.5 (±9.4) ^‡^
To celebrate a special occasion	24.0 (±8.4)	14.6 (±6.9)	35.2 (±9.4) ^γ^	37.2 (±9.5) ^†^
To get drunk	23.2 (±8.3)	8.0 (±5.3)	45.6 (±9.8) ^γ^	31.2 (±9.1) ^†‡^
I received the drink from someone else (and did not want to refuse it)	13.6 (±6.7)	7.0 (±5.0)	23.4 (±8.3) ^γ^	16.8 (±7.3) ^†‡^
Because others drink it as well	10.2 (±5.9)	4.8 (±4.2)	17.6 (±7.5) ^γ^	14.9 (±7.0) ^†^
It feels like it reduces the negative effects of alcohol	9.2 (±5.7)	6.9 (±5.0)	10.8 (±6.1) ^γ^	16.8 (±7.3) ^†‡^
To make me happy	7.9 (±5.3)	5.0 (±4.3)	10.5 (±6.0) ^γ^	14.1 (±6.8) ^†^
It feels like I can drink more alcohol	7.9 (±5.3)	5.6 (±4.5)	10.0 (±5.9) ^γ^	14.0 (±6.8) ^†^
To prevent getting drunk	3.0 (±5.3)	3.8 (±3.7)	1.0 (±2.0) ^γ^	5.9 (±4.6) ^‡^
To sober up	2.8 (±3.2)	2.9 (±3.3)	1.6 (±2.5)	2.6 (±3.1)
To reduce next-day hangover effects	2.1 (±2.8)	2.4 (±3.0)	1.5 (±2.4)	2.3 (±2.9)
I felt sad	1.1 (±2.0)	0.3 (±1.0)	2.3 (±2.9) ^γ^	1.8 (±2.6)

Percentage and 95% confidence interval (between brackets) of participants that endorsed each item are shown. Significant differences (adjusted *p* value < 0.05) between the Netherlands and the UK by ^γ^, the Netherlands and Australia by ^†^, and between the UK and Australia by ^‡^.

**Table 3:** Past month alcohol consumption of AMED and AO consumers on usual AO occasions (between-group comparisons).

*Between-Group Comparisons*	Overall		The Netherlands		UK		Australia	
Group	AMED Group	AO Group	AMED Group	AO Group	AMED Group	AO Group	AMED Group	AO Group
N	2202	4608	1239	3185	729	797	234	629
**Usual drinking occasions**								
Number of alcoholic drinks	6.9 (4.9)	4.6 (3.5) *	6.0 (3.9)	4.1 (3.1) *	8.9 (6.0) ^γ^	6.4 (4.1) *^γ^	6.4 (3.8) ^†‡^	4.6 (3.3) * ^‡^
Number of drinking days (alcohol)	8.3 (6.1)	7.3 (6.1) *	9.2 (6.4)	7.9 (6.3) *	7.1 (5.3) ^γ^	6.1 (4.7) *^γ^	7.2 (6.2) ^†^	5.7 (5.8) *^†‡^
Days got drunk	2.8 (3.4)	1.5 (2.4) *	1.9 (2.7)	1.0 (1.9) *	4.0 (3.8) ^γ^	2.8 (3.0) *^γ^	3.4 (4.0) ^†‡^	2.0 (2.9) *^†‡^
Binge drinking days (>4/5 alcoholic drinks)	4.7 (4.6)	2.9 (3.8) *	4.8 (4.8)	2.9 (3.9) *	4.7 (4.4)	3.3 (3.4) *^γ^	4.1 (4.1) ^†‡^	2.5 (3.6) *^†‡^
**Past month heaviest drinking occasion**								
Number of alcoholic drinks	11.3 (7.4)	7.8 (6.1) *	10.7 (6.7)	7.7 (6.0) *	12.8 (8.5) ^γ^	9.4 (6.4) *^γ^	9.5 (6.1) ^†‡^	6.7 (5.6) *^‡^
Hours of drinking	5.9 (3.1)	5.1 (3.0) *	6.0 (3.1)	5.1 (3.1) *	5.8 (3.0)	5.2 (2.8) *	5.6 (3.2) ^†^	4.7 (3.1) *^†‡^

Mean and standard deviation (between brackets) are shown. Significant differences (*p* < 0.05) between AMED and AO are indicated by *. Significant differences (adjusted *p*-value < 0.05) between the Netherlands and the UK are indicated by ^γ^, between the Netherlands and Australia by ^†^, and between the UK and Australia by ^‡^. Note: sample sizes may differ from those reported in Table 1 as not all participants reported past month AMED consumption.

**Table 4:** Alcohol consumption of AMED consumers on usual AMED and AO occasions (within-subject comparisons).

Within-Subject Comparisons	Overall		The Netherlands		UK		Australia	
N	2202		1239		729		234	
**Usual drinking occasions**	**AMED**	**AO**	**AMED**	**AO**	**AMED**	**AO**	**AMED**	**AO**
Number of alcoholic drinks	5.6 (4.1)	6.9 (4.9) *	5.4 (3.7)	6.0 (3.9) *	6.0 (4.9)	9.0 (6.1) *^γ^	5.1 (3.7) ^‡^	5.9 (3.7) *^‡^
Number of energy drinks	1.8 (1.8)	0.0 (0.0) *	1.6 (1.1)	0.0 (0.0) *	2.3 (2.5) ^γ^	0.0 (0.0) *	1.8 (1.6) ^†‡^	0.0 (0.0) *
Number of drinking days (alcohol)	1.7 (2.2)	8.3 (6.1) *	1.4 (1.8)	9.2 (6.4) *	2.3 (2.8) ^γ^	7.0 (5.3) *^γ^	1.5 (2.1) ^‡^	7.3 (6.1) *^†^
Days got drunk	1.1 (2.0)	2.7 (3.2) *	0.5 (1.0)	4.7 (4.7) *	2.1 (2.7) ^γ^	4.0 (3.8) *^γ^	1.2 (1.8) ^†‡^	3.3 (3.8) *^†‡^
Binge drinking days (>4/5 alcoholic drinks)	1.3 (2.3)	4.6 (4.6) *	0.9 (1.7)	4.7 (4.7) *	2.1 (2.9) ^γ^	4.7 (4.4) *	1.3 (2.0) ^†‡^	4.1 (4.0) *^‡^

Mean and standard deviation (between brackets) are shown. Significant differences (*p* < 0.05) between AMED and AO are indicated by *. Significant differences (adjusted *p*-value < 0.05) between the Netherlands and the UK are indicated by ^γ^, between the Netherlands and Australia by ^†^, and between the UK and Australia by ^‡^.

**Table 5:** Alcohol consumption of AMED consumers on their past month heaviest AMED and AO occasion (within-subject comparisons).

Within-Subject Comparisons	Overall		The Netherlands		UK		Australia	
N	1477		816		501		160	
**Past month heaviest drinking occasion**	**AMED**	**AO**	**AMED**	**AO**	**AMED**	**AO**	**AMED**	**AO**
Number of alcoholic drinks	7.4 (6.8)	12.1 (7.3) *	6.5 (5.9)	11.3 (6.6) *	8.7 (7.6) ^γ^	14.1 (8.2) *^γ^	7.5 (6.2)	9.9 (6.0) *^‡^
Number of energy drinks	2.6 (2.2)	0.0 (0.0) *	2.3 (1.9)	0.0 (0.0) *	3.1 (2.3) ^γ^	0.0 (0.0) *	2.5 (2.3) ^‡^	0.0 (0.0) *
Hours of drinking	5.3 (2.9)	6.2 (3.0) *	5.2 (2.9)	6.4 (3.0) *	5.5 (2.6)	6.2 (2.8) *	5.3 (3.3)	5.7 (3.1) *

Mean and standard deviation (between brackets) are shown. Significant differences (*p* < 0.05) between AMED and AO are indicated by *. Significant differences (adjusted *p*-value < 0.05) between the Netherlands and the UK are indicated by ^γ^, and between the UK and Australia by ^‡^. No significant differences were found between the Netherlands and Australia. Note: sample sizes may differ from those reported in Table 1 as not all participants reported past month AMED consumption on their past month heaviest drinking occasion.

**Table 6:** Alcohol related consequences.

Alcohol-Related Consequence	Overall		The Netherlands		UK		Australia	
	AMED	AO	AMED	AO	AMED	AO	AMED	AO
I have had a hangover (headache, sick stomach) the morning after I had been drinking.	60.5 (±9.6)	82.4 (±7.5) *	47.0 (±9.8)	77.6 (±8.2) *	77.1 (±8.2) ^γ^	86.2 (±6.8) *^γ^	56.0 (±9.7) ^†‡^	79.3 (±7.9) *^‡^
I have had less energy or felt tired because of my drinking.	39.8 (±9.6)	67.8 (±9.2) *	29.7 (±9.0)	62.8 (±9.5) *	50.3 (±9.8) ^γ^	69.6 (±9.0) *^γ^	35.0 (±9.3) ^†‡^	60.3 (±9.6) *^‡^
While drinking, I have said or done embarrassing things.	33.5 (±9.3)	49.1 (±9.8) *	17.4 (±7.4)	34.8 (±9.3) *	61.6 (±9.5) ^γ^	73.5 (±8.7) *^γ^	39.3 (±9.6) ^†‡^	55.6 (±9.7) *^†‡^
I have had felt very sick to my stomach or thrown up after drinking.	33 (±9.2)	49.3 (±9.8) *	19.8 (±7.8)	37.1 (±9.5) *	49.2 (±9.8) ^γ^	60.8 (±9.6) *^γ^	32.5 (±9.2) ^†‡^	56.4 (±9.7) *^†‡^
I’ve not been able to remember large stretches of time while drinking heavily.	31.4 (±9.1)	47.5 (±9.8) *	17.8 (±7.5)	35.4 (±9.4) *	50.3 (±9.8) ^γ^	60.6 (±9.6) *^γ^	24.4 (±8.4) ^†‡^	45.7 (±9.8) *^†‡^
I have taken foolish risks when I have been drinking.	28.6 (±8.9)	39.5 (±9.6) *	15.2 (±7.0)	27.0 (±8.7) *	45.0 (±9.8) ^γ^	51.1 (±9.8) *^γ^	42.6 (±9.7) ^†‡^	49.6 (±9.8) *^†‡^
I have not gone to work or missed classes at school because of drinking, a hangover or illness caused by drinking.	24.4 (±8.4)	34.7 (±9.3) *	12.4 (±6.5)	24.1 (±8.4) *	41.4 (±9.7) ^γ^	47.7 (±9.8) *^γ^	17.9 (±7.5) ^†‡^	31.5 (±9.1) *^‡^
I often have ended up drinking on nights when I had planned not to drink.	20.3 (±7.9)	39.9 (±9.6) *	10.3 (±6.0)	27.7 (±8.8) *	32.4 (±9.2) γ	52.8 (±9.8) *^γ^	21.8 (±8.1) ^†‡^	42.7 (±9.7) *^†‡^
I have found that I needed larger amounts of alcohol to feel any effect, or that I could no longer get high or drunk on the amount that used to get me high or drunk.	18.5 (±7.6)	25.7 (±8.6) *	11.0 (±6.1)	16.4 (±7.3) *	26.7 (±8.7) ^γ^	34.8 (±9.3) *^γ^	18.8 (±7.7) ^†‡^	28.6 (±8.9) *^†‡^
When drinking, I have done impulsive things I regretted later.	18.3 (±7.6)	26.6 (±8.7) *	10.3 (±6.0)	19.7 (±7.8) *	33.3 (±9.2) ^γ^	38.9 (±9.6) *^γ^	18.8 (±7.7) ^†‡^	28.2 (±8.8) *^†‡^
The quality of my work or school work has suffered because of my drinking.	15.3 (±7.1)	27.7 (±8.8) *	10.5 (±6.0)	22.7 (±8.2) *	19.0 (±7.7) ^γ^	28.9 (±8.9) *^γ^	15.8 (±7.1) ^†‡^	28.6 (±8.9) *^†‡^
My drinking has gotten me into sexual situations I later regretted.	14.9 (±7.0)	23.2 (±8.3) *	6.1 (±4.7)	13.7 (±6.7) *	26.5 (±8.7) ^γ^	33.8 (±9.3) *^γ^	15.0 (±7.0) ^†‡^	23.5 (±8.3) *^†‡^
I have often found it difficult to limit how much I drink.	14.3 (±6.9)	19.1 (±7.7) *	5.0 (±4.3)	8.3 (±5.4) *	26.5 (±8.7) ^γ^	31.4 (±9.1) *^γ^	15.4 (±7.1) ^†‡^	25.2 (±8.5) *^†‡^
I have felt badly about myself because of my drinking.	13.6 (±6.7)	20.6 (±7.9) *	7.4 (±5.1)	14.0 (±6.8) *	20.9 (±8.0) ^γ^	25.8 (±8.6) *^γ^	13.2 (±6.6) ^†‡^	23.1 (±8.3) *^†‡^
I have spent too much time drinking.	13.0 (±6.6)	20.5 (±7.9) *	5.6 (±4.5)	10.9 (±6.1) *	21.9 (±8.1) ^γ^	31.1 (±9.1) *^γ^	15.0 (±7.0) ^†‡^	24.4 (±8.4) *^†‡^
I have become very rude, obnoxious, or insulting after drinking.	11.8 (±6.3)	15.0 (±7.0) *	3.8 (±3.7)	5.9 (±4.6) *	21.7 (±8.1) ^γ^	26.0 (±8.6) *^γ^	14.5 (±6.9) ^†‡^	18.4 (±7.6) ^†‡^
I have been overweight because of drinking.	11.1 (±6.2)	16.7 (±7.3) *	6.9 (±5.0)	13.6 (±6.7) *	15.4 (±7.1) ^γ^	19.4 (±7.8) *^γ^	10.3 (±6.0) ^†‡^	9.8 (±5.8) ^‡^
I have passed out from drinking.	10.1 (±5.9)	14.1 (±6.8) *	2.3 (±2.9)	1.9 (±2.7)	19.4 (±7.8) ^γ^	26.5 (±8.7) *^γ^	15.4 (±7.1) ^†‡^	30.3 (±9.0) *^†‡^
I have neglected my obligations to family, work, or school because of drinking.	9.6 (±5.8)	13.5 (±6.7) *	5.6 (±4.5)	7.7 (±5.2) *	14.3 (±6.9) ^γ^	19.5 (±7.8) *^γ^	8.1 (±5.3) ^†‡^	14.1 (±6.8) *^†‡^
My physical appearance has been harmed by my drinking.	9.1 (±5.6)	12.5 (±6.5) *	4.6 (±4.1)	6.1 (±4.7) *	16.5 (±7.3) ^γ^	22.6 (±8.2) *^γ^	12.0 (±6.4) ^†^	17.1 (±7.4) *^†‡^
My drinking has created problems between myself and my boyfriend/girlfriend/spouse, parents, or other near relatives.	8.1 (±5.3)	12.4 (±6.5) *	4.5 (±4.1)	7.1 (±5.0) *	11.5 (±6.3) ^γ^	17.7 (±7.5) *^γ^	9.0 (±5.6) ^†‡^	13.2 (±6.6) ^†‡^
I have woken up in an unexpected place after heavy drinking.	7.9 (±5.3)	12.7 (±6.5) *	3.3 (±3.5)	6.8 (±4.9) *	16.3 (±7.2) ^γ^	22.9 (±8.2) *^γ^	8.5 (±5.5) ^†‡^	14.5 (±6.9) *^†‡^
I have driven a car when I knew I had too much to drink to drive safely.	5.7 (±4.5)	8.5 (±5.5) *	2.8 (±3.2)	5.0 (±4.3) *	7.5 (±5.2) ^γ^	9.5 (±5.7) *^γ^	9.8 (±5.8) ^†‡^	15.4 (±7.1) *^†‡^
I have felt like I needed a drink after I’d gotten up (that is, before breakfast).	4.0 (±3.8)	5.1 (±4.3) *	2.3 (±2.9)	2.3 (±2.9)	4.1 (±3.9) ^γ^	7.6 (±5.2) *^γ^	7.7 (±5.2) ^†‡^	8.6 (±7.1) ^†‡^
Total BYAACQ score	4.4 (4.6)	6.6 (4.9) *	2.6 (3.4)	4.9 (3.8) *	7.1 (5.1) ^γ^	9.0 (5.4) *^γ^	6.3 (4.4) ^†^	8.7 (4.8) *^†^

The percentage of endorsement and 95% confidence interval (between brackets) are listed for each item. Mean and standard deviation (between brackets) are shown for the total BYAACQ score. Significant differences (*p* < 0.05) between AMED and AO are indicated by *. Significant differences (adjusted *p*-value < 0.05) between the Netherlands and the UK are indicated by ^γ^, between the Netherlands and Australia by ^†^, and between the UK and Australia by ^‡^.

**Table 7:** Demographics.

Demographics	Men (N = 2405)		Women (N = 4476)	
	AMED	AO	AMED	AO
N	903	1502	1299	3177
Age (years)	21.2 (2.5)	22.3 (3.1) *	21.3 (2.6)	22.0 (3.3) *^S^
Height (m)	1.82 (0.1)	1.83 (0.1)	1.69 (0.1) ^†^	1.69 (0.1) ^S^
Weight (kg)	79.1 (15.3)	76.7 (12.5) *	65.3 (12.4) ^†^	64.1 (10.6) *^S^
Current smoker (%)	21.5 (±8.1)	12.5 (±6.5) *	21.9 (±8.1)	10.0 (±5.9) *
Past year illicit drug use (%)	40.9 (±9.6)	26.3 (±8.6) *	28.3 (±8.8) ^S^	18.3 (±7.6) *^S^
Medication use (%)	11.2 (±6.2)	10.6 (±6.0)	28.9 (±8.9) ^S^	28.1 (±8.8) *^S^
Age first consumed alcohol	13.9 (2.5)	14.5 (2.4) *	14.1 (2.2)	14.5 (2.3) *
Age regular alcohol use	16.7 (1.7)	17.2 (1.9) *	16.8 (1.7)	17.4 (2.1) *^S^
RT18 total risk-taking score	8.8 (3.9)	6.8 (3.9) *	7.2 (4.1) ^S^	5.8 (3.7) *^S^
RT18 Factor 1 (risk-taking)	5.8 (2.5)	4.6 (2.7) *	4.3 (2.6) ^S^	3.5 (2.5) *^S^
RT18 Factor 2 (risk assessment)	3.0 (2.3)	2.3 (2.0) *	2.9 (2.2)	2.3 (2.0) *

Percentages and 95% confidence interval (between brackets) or mean and standard deviation (SD, between brackets) are shown. Significant differences between AMED and AO (*p* < 0.05) are indicated by *, significant differences between men and women (*p* < 0.05) are indicated by ^S^, between the Netherlands and Australia by ^†^. Abbreviations: AMED = alcohol mixed with energy drink, AO = alcohol only.

**Table 8:** Motives for alcohol mixed with energy drink (AMED) consumption among men and women.

Motives	Men	Women
I like the taste	74.3 (±8.6)	74.5 (±8.5)
I wanted to drink something else	37.2 (±9.5)	28.1 (±8.8) ^S^
To get drunk	29.9 (±9.0)	18.6 (±7.6) ^S^
To celebrate a special occasion	20.0 (±7.8)	17.7 (±7.5)
I received the drink from someone else (and did not want to refuse it)	15.1 (±7.0)	12.5 (±6.5) ^S^
It feels like it reduces the negative effects of alcohol	12.8 (±6.5)	6.8 (±4.9) ^S^
Because others drink it as well	12.4 (±6.5)	8.7 (±5.5) ^S^
It feels like I can drink more alcohol	10.0 (±5.9)	6.5 (±4.8) ^S^
To make me happy	7.3 (±5.1)	5.4 (±4.4) ^S^
To prevent getting drunk	3.5 (±3.6)	2.7 (±3.2)
To sober up	2.8 (±3.2)	1.3 (±2.2) ^S^
To reduce next-day hangover effects	2.8 (±3.2)	1.6 (±2.5) ^S^
I felt sad	0.9 (±1.9)	1.2 (±2.1)

Percentage and 95% confidence interval (between brackets) of subjects that endorsed each item are shown. Significant differences (*p* < 0.05) between men and women are indicated by ^S^.

**Table 9:** Past month alcohol consumption of AMED and AO consumers on usual AO occasions (between-group comparisons).

Between-Group Comparisons	Men		Women	
Group	AMED Group	AO Group	AMED Group	AO Group
N	903	1471	1299	3138
**Usual drinking occasions**				
Number of alcoholic drinks	8.6 (5.6)	5.9 (4.4) *	5.8 (3.8) ^S^	4.0 (2.7) *^S^
Number of drinking days (alcohol)	9.1 (6.4)	8.9 (6.8) *	7.7 (5.9) ^S^	6.5 (5.5) *^S^
Days got drunk	3.4 (3.7)	2.0 (2.7) *	2.3 (2.9) ^S^	1.2 (2.2) *^S^
Binge drinking days (>4/5 alcoholic drinks)	5.8 (5.1)	4.2 (4.6) *	3.9 (4.0) ^S^	2.3 (3.1) *^S^
**Past month heaviest drinking occasion**				
Number of alcoholic drinks	14.3 (8.2)	10.9 (7.6) *	9.1 (5.7) ^S^	6.4 (4.6) *^S^
Hours of drinking	6.3 (3.2)	5.7 (3.5) *	5.6 (2.8) ^S^	4.8 (2.8) *^S^

Mean and standard deviation (between brackets) are shown. Significant differences (*p* < 0.05) between AMED and AO are indicated by *, significant differences between men and women are indicated by ^S^. Note: sample sizes may differ from those reported in Table 1 as not all participants reported past month AMED consumption.

**Table 10:** Alcohol consumption of male and female AMED consumers on usual AMED and AO occasions (within-subject comparisons).

Within-Subject Comparisons	Men		Women	
N	903		1299	
**Usual drinking occasions**	**AMED**	**AO**	**AMED**	**AO**
Number of alcoholic drinks	6.9 (4.7)	8.6 (5.6) *	4.6 (3.0) ^S^	5.7 (3.8) *^S^
Number of energy drinks	2.0 (1.8)	0.0 (0.0) *	1.6 (1.1) ^S^	0.0 (0.0) *^S^
Number of drinking days (alcohol)	2.0 (2.5)	9.1 (6.4) *	1.5 (1.9) ^S^	7.7 (5.9) *^S^
Days got drunk	1.4 (2.2)	3.4 (3.7) *	0.8 (1.6) ^S^	2.3 (2.9) *^S^
Binge drinking days (>4/5 alcoholic drinks)	1.7 (2.5)	5.8 (5.1) *	1.0 (1.8) ^S^	3.9 (4.0) *^S^
**Past month heaviest drinking occasion**	**AMED**	**AO**	**AMED**	**AO**
Number of alcoholic drinks	7.2 (7.7)	14.3 (8.2) *	4.1 (5.1) ^S^	9.1 (5.7) *^S^
Number of energy drinks	2.3 (2.5)	0.0 (0.0) *	1.6 (1.8) ^S^	0.0 (0.0) *^S^
Hours of drinking	4.6 (3.4)	6.3 (3.24) *	3.8 (3.2) ^S^	5.6 (2.8) *^S^

Mean and standard deviation (between brackets) are shown. Significant differences (*p* < 0.05) between AMED and AO are indicated by *. Significant differences (*p* < 0.05) between men and women are indicated by ^S^.

**Table 11:** Alcohol-related consequences reported by men and women.

Alcohol-Related Consequences	Men		Women	
	AMED	AO	AMED	AO
I have had a hangover (headache, sick stomach) the morning after I had been drinking.	67.0 (±9.2)	86.6 (±6.7) *	55.9 (±9.7) ^S^	79.4 (±7.9) *^†S^
I have had less energy or felt tired because of my drinking.	42.9 (±9.7)	68.7 (±9.1) *	37.5 (±9.5) ^S^	67.2 (±9.2) *
While drinking, I have said or done embarrassing things.	38.4 (±9.5)	51.8 (±9.8) *	30.1 (±9.0) ^S^	47.2 (±9.8) *^S^
I have had felt very sick to my stomach or thrown up after drinking.	37.8 (±9.5)	53.7 (±9.8) *	29.6 (±8.9) ^S^	46.2 (±9.8) *^S^
I’ve not been able to remember large stretches of time while drinking heavily.	39.7 (±9.6)	55.0 (±9.8) *	25.5 (±8.5) ^S^	42.2 (±9.7) *^S^
I have taken foolish risks when I have been drinking.	36.9 (±9.5)	48.5 (±9.8) *	22.7 (±8.2) ^S^	33.2 (±9.2) *^S^
I have not gone to work or missed classes at school because of drinking, a hangover or illness caused by drinking.	31.3 (±9.1)	41.6 (±9.7) *	19.5 (±7.8) ^S^	30.0 (±9.0) *^S^
I often have ended up drinking on nights when I had planned not to drink.	24.2 (±8.4)	45.0 (±9.8) *	17.6 (±7.5) ^S^	36.4 (±9.4) *^S^
I have found that I needed larger amounts of alcohol to feel any effect, or that I could no longer get high or drunk on the amount that used to get me high or drunk.	24.7 (±8.5)	34.2 (±9.3) *	14.2 (±6.8) ^S^	19.8 (±7.8) *^S^
When drinking, I have done impulsive things I regretted later.	24.9 (±8.5)	33.1 (±9.2) *	13.7 (±6.7) ^S^	22.1 (±8.1) *^S^
The quality of my work or school work has suffered because of my drinking.	21.7 (±8.1)	34.1 (±9.3) *	10.7 (±6.1) ^S^	23.2 (±8.3) *^S^
My drinking has gotten me into sexual situations I later regretted.	20.8 (±8.0)	30.0 (±9.0) *	10.7 (±6.1) ^S^	18.4 (±7.6) *^S^
I have often found it difficult to limit how much I drink.	17.3 (±7.4)	23.4 (±8.3) *	12.2 (±6.4) ^S^	16.2 (±7.2) *^S^
I have felt badly about myself because of my drinking.	15.8 (±7.1)	23.7 (±8.3) *	12.0 (±6.4) ^S^	18.3 (±7.6) *^S^
I have spent too much time drinking.	17.8 (±7.5)	28.7 (±8.9) *	9.6 (±5.8) ^S^	14.8 (±7.0)*^S^
I have become very rude, obnoxious, or insulting after drinking.	16.5 (±7.3)	21.5 (±8.1) *	8.4 (±5.4) ^S^	10.4 (±6.0) *^S^
I have been overweight because of drinking.	11.6 (±6.3)	18.6 (±7.6) *	10.8 (±6.1) ^S^	15.4 (±7.1) *^S^
I have passed out from drinking.	12.8 (±6.5)	17.7 (±7.5) *	8.1 (±5.3) ^S^	11.3 (±6.2) *^S^
I have neglected my obligations to family, work, or school because of drinking.	12.8 (±6.5)	17.8 (±7.5) *	7.4 (±5.1) ^S^	10.5 (±6.0) *^S^
My physical appearance has been harmed by my drinking.	13.3 (±6.7)	16.1 (±7.2) *	6.1 (±4.7) ^S^	9.3 (±5.7) *^S^
My drinking has created problems between myself and my boyfriend/girlfriend/spouse, parents, or other near relatives.	10.2 (±5.9)	15.4 (±7.1) *	6.6 (±4.9) ^S^	10.4 (±6.0) *^S^
I have woken up in an unexpected place after heavy drinking.	13.4 (±6.7)	21.6 (±8.1) *	4.1 (±3.9) ^S^	6.5 (±4.8) *^S^
I have driven a car when I knew I had too much to drink to drive safely.	9.0 (±5.6)	12.6 (±6.5) *	3.4 (±3.6) ^S^	5.6 (±4.5) ^S^
I have felt like I needed a drink after I’d gotten up (that is, before breakfast).	5.1 (±4.3)	7.1 (±5.0) *	3.1 (±3.4) ^S^	3.7 (±3.7) ^S^
Total BYAACQ score	5.4 (5.1)	7.8 (5.1) *^†^	3.6 (4.1) ^S^	5.7 (4.5) *^S^

The percentage of endorsement and 95% confidence interval (between brackets) are listed for each item. Mean and standard deviation (between brackets) are shown for the total BYAACQ score. Significant differences (*p* < 0.05) between AMED and AO are indicated by *. Significant differences (*p* < 0.05) between men and women are indicated by ^S^, between the Netherlands and Australia by ^†^.

## Data Availability

The data are available on request from the corresponding author.
